# Medico-Chirurgical Transactions

**Published:** 1822-09-01

**Authors:** 


*Medico-Chirurgical Transactions.* Vol. XII. Part I. 8vo, pp. 254, with plates. 1822.

This first part of the twelfth volume is certainly superior to some late parts of preceding volumes; and sincerely do we hope that the prize, which is now offered for the best paper annually, may prove an attraction for communications of sterling merit. " In selecting papers, say the council, for the prize, the choice shall not be confined to those written by members only, but it shall extend to all papers which shall have been read to the society." We have no doubt that the council are perfectly aware of the responsibility which they thus incur. Not only is their impartiality, but their judgment also is at stake, and the public are rigidly just censors on all such occasions. With due deference, we humbly beg leave to question the policy of the council in resolving that the Society's transactions shall appear at stated epochs, viz. on the 1st of July, and 1st of February, of each year. They thus render their volume a kind of stage coach, which, by being obliged to start at fixed periods, is often obliged to run with scanty fares. This, indeed, is now well known to be the great draw-back on all periodical publications of original papers, and we are somewhat surprized that in multiplicity of council, where there is, of necessity, the maximum of wisdom, this obstacle should not have been taken into consideration. The Medico-Chirurgical Society should recollect that their transactions are now viewed, in foreign realms at least, as a tolerable scale or index of the state of medical and surgical science in this country. Surely, then, they should not voluntarily impose on themselves laws which are but too well calculated to draw a shade over their former lustre. But we shall not pursue the subject farther, as we are well convinced that "corporate bodies," (a term, by the bye, which we have always looked upon as a piece of tautology, for can bodies be *incorporate?*) will very rarely listen to the advice of individuals, however conscious they may be of the propriety of the advice.

The volume before us contains twenty papers of very unequal extent and value.

*Art.* I. Dr. Marshall Hall relates the cases of four children who had attempted, by mistake, to drink boiling water from the spout of a tea-kettle. "The effects of such accident," says he, "are not, as might be supposed, *à priori*, inflammation of the œsophagus and stomach, but of the glottis and larynx, resembling croup"—constituting instances where the operation of laryngotomy or tracheotomy may be necessary to prevent impending suffocation.* We agree with our author, that it is hardly probable that any of the boiling water reaches the stomach. Its presence must throw the muscular fibres of the pharynx and œsophagus into such spasmodic contraction, as will effectually prevent its further progress in that direction.

* When we consider that the fauces, œsophagus, and stomach, are accustomed to have hot and solid substances daily applied to them, while the membrane lining the passage to the lungs is only accustomed to an aerial current, we cannot wonder that, in such accidents as the present, the larynx and glottis should be the principal sufferers. The first inspiration, under such circumstances, would convey burning steam to the tender and delicate tissue covering the air passages, and the severe consequences may be readily imagined.—*Rev.*

"Of the four patients whose cases are about to be given, one recovered from imminent suffocation immediately after violent screaming; two died from suffocation—one 10, the other 17 hours after the accident; the fourth was completely relieved by the operation of tracheotomy—survived 34 hours, but died, exhausted by the irritation produced by the primary affection." 2.

The first little patient, three years of age, had no assistance for three or four hours after the accident, during which interim augmenting difficulty of breathing came on. A mixture of oil and syrup was recommended; but the dyspnœa continuing to increase, the little patient was bled from the jugular vein, without any relief. Suffocation now threatened, and leeches were ordered. But the sight of the leeches caused the patient to scream violently, and they could not be applied. The dyspnœa now subsided, and she was well in a week. The parents supposed, and with no mean degree of probability, that the screaming ruptured the vesicles which impeded the breathing and thus proved the unexpected means of cure.

The second child was but two years of age, and in four hours after the accident began to labour and rattle in breathing. He died from suffocation about seventeen hours from the receipt of the injury. The little patient had been bled, and took oily linctuses, but without benefit.

The third patient, of the same age, shared a similar fate in ten hours from the accident.

The fourth patient was two and half years of age, and our author saw the child five hours after the injury, when she was breathing with difficulty, and with a hoarse croupy sound. She was able to swallow without manifest pain or coughing. The tongue and all the internal parts of the mouth were blanched and blistered—pulse frequent—dyspnœa increasing. To prevent impending suffocation, tracheotomy was performed twelve hours after the accident. The relief was immediate. The little patient sat up, played, looked cheerful. The voice was, of course, extinct—the breathing free through the orifice. In six hours the dyspnœa had considerably returned—the face was pale, and the child appeared to be dying. In a few hours, however, the child rallied, and the dyspnœa had lessened. Next day the little patient was worse, and apparently sinking. She died at 2 o'clock, or about 34 hours after the operation.

"On dissection, there was observed a swollen, blistered, and corrugated state of the epiglottis; and a similar state of the posterior fauces, tongue, and internal mouth. There was a little mucus in the larynx, but no perceptible morbid condition of the œsophagus or stomach. There was no inflammation of the trachea, not even near the orifice made by the operation." 7.

To the above cases Mr. Stanley, of Guy's Hospital, has appended the particulars of two analogous cases—one, from the private practice of Mr. Gillman of Highgate—the other from the records of Bartholomew's Hospital.

Mr. Gillman's patient was a little girl, of three or four years of age, who drank, or attempted to drink, boiling water from the spout of a tea-kettle, at seven o'clock in the evening. Mr. G. saw the child in an hour afterwards. It had been vomiting violently in the interval—saliva flowed copiously from its mouth—pulse small, quick, and feeble. Oil of almonds with gruel, and some tincture of opium were ordered every four hours, and an aperient next morning. By that time, however, the child was much worse—there was great difficulty of breathing, vomiting, and short cough. Leeches were applied to the trachea. The poor child struggled through a painful existence till the second morning, or 38th hour from the accident. On inspection the whole interior of the mouth, fauces, pharynx, and principal portion of œsophagus "presented the usual appearances of a scald." The stomach was but little affected. The lining of the trachea was much inflamed, and on different parts depositions of coagulable lymph were observed.

The case in Bartholomew's was a child three years old, who lived twelve hours after the accident. In this case the symptoms seemed to indicate disorder of the brain, as sunk countenance, gloominess of aspect, and complete prostration of the vital powers.

"Upon examination of the body, a slight effusion of transparent fluid was found between the tunica arachnoidea and the pia mater, and into the cellular texture of the latter. About three drachms of a similar fluid were found in the lateral ventricles of the brain. The vessels of the brain were not preternaturally turgid. There was a slight redness and tumefaction in the mucous membrane of the pharynx, and upper part of the larynx, above the opening of the glottis. The glottis was of its natural diameter; the morbid appearances were just sufficient to show that irritation had existed in the parts. The trachea, œsophagus, and stomach were of a healthy appearance." P. 11.

From the facts which are here presented to us, we should be led to conclude that, wherever the dyspnœa was the pressing symptom, and it was so in five cases out of six, laryngotomy is the only measure which promises success. But it should be performed early, and the opening should be kept perfectly pervious till all danger is over. Would not ice-creams kept constantly dissolving in the mouth be more soothing, and perhaps more salutary, than syrups and oils?

*Art.* 2. This is a case of ligature of the subclavian artery by Charles Mayo, Esq. Surgeon to the Winchester Hospital. We regret to say that it was not successful—though it deserved success.

The patient was a man 38 years of age, who was admitted in March 1821, with a pulsating tumour below the left clavicle, which was readily ascertained to be aneurism of the axillary artery. The patient had perceived the tumour about four months, and it was always pulsatory. The pulsation was now (March 18th) very strong, and the artery above the clavicle not easily compressible. The tumour seemed rather to overlap than elevate the bone. The pulse at the wrist was the same as in the other arm. He complained of severe pain and irritation in the breast, shoulder, and arm. Having been bled, purged, and an opiate given at bed time, he was operated on next day, the 19th March. We shall give the steps of the operation in the author's own words.

"The man was laid on the operating table, with his shoulders a little raised, and his legs hanging over the table, and resting on a chair. I made a transverse incision, about two inches and a half in length, along the upper edge of the clavicle, and from the middle of this incision I made another directly upwards along the outer margin of the sterno-cleido-mastoideus muscle; I then dissected back the triangular flaps, thereby exposing the edge of the mastoid muscle, and the omo-hyoideus, which crossed the upper angle of the wound. In the space between the latter muscle and the clavicle, I now felt the artery with the end of my finger, and then proceeded to expose it fairly by removing the cellular tissue which covered it. This cellular tissue was partly divided by a few cautious touches of the scalpel, and partly detached from the vessel by the handle of the instrument. I next directed my assistant to draw the nerves a little outwards with a hook, and then, passing my finger into the bottom of the wound, I felt the artery distinctly upon the rib, and, pressing upon it, stopped the pulsations in the tumour. I touched the cellular tissue cautiously on each side of the artery with the edge of the scalpel, but still could not raise it from the rib with my nail; it seemed firmly attached in its situation. I tried to pass probes and other instruments beneath the vessel, but ineffectually. At length, I insinuated the tip of Desaut's elastic needle under the outer-side of the artery, and, holding the canula firm upon the rib, I desired the assistant to press down the stilet, which caused the needle to pass under the artery; but I had some difficulty in bringing its extremity up on the other side, as it constantly hitched in the neighbouring fibres of the scalenus and mastoid muscles, the great flexibility of the needle allowing its course to be altered by the slightest obstacles; at last, by catching the point with my nail, I was able to guide it round the vessel, while my assistant pressed it forward; I next threaded the needle with a strong round ligature, and cautiously withdrew it into the canula; then, bringing the double ligature under the artery, I cut it from the needle. The two portions of ligatnre were now separated from each other as much as possible, and one was tied; then, with the assistance of Mr. Ramsden's iron instruments for tightening the ligature, the knot was drawn, the inner coats of artery were sensibly divided, and the pulsations in the tumour ceased; a second knot was then made, and tightened in the same manner. I now attempted to withdraw the loose portion of ligature, but found that it was so connected with that which had been tied, as not to allow of its being withdrawn without force; therefore I let the whole remain. The ligatures were brought out at the lower part of the wound, the edges of which were approximated by strips of plaster, and over this a light compress was confined." 15.

The hæmorrhage was trifling, and the patient passed a good night. For the diary of the case, from the day of operation till the 25th March, we must refer to the volume. We may remark, however, that the pulsation returned in the tumour, and even at the wrist, the day after the operation. On the sixth day, arterial hæmorrhage occurred to the amount of a pint. The pulsation of the tumour now ceased, and the size became diminished to one-third its original size. There was no more hæmorrhage till the 29th of March; but on the 28th there were shivering fits, with delirium, fever, and other unfavourable symptoms. On the 29th there was hæmorrhage from the wound, which was the case on the 30th and 31st, but not to such extent. On the latter day he died.

"April 1st.—*Dissection.*—The aneurismal sac was exposed by the reflexion of the pectoralis major and minor muscles; its lower boundary extended to the inferior margins of the third rib; and above, it extended beneath the clavicle to the first rib; laterally it reached from the axilla to the sternum. The axillary vein was found exterior to, and firmly united to the sac, at its inferior part. Three or four large nerves of the axillary plexus were stretched over the middle of the sac. In the further progress of the examination, the following circumstances were noticed:—The aorta appeared thinner than usual, and the carotids were in some parts so thin as to be transparent. The left subclavian artery was completely divided at the part where it had been tied, and the two portions of the vessel were separated about a quarter of an inch from each other, the ligature being retained by a few cellular threads in the midspace between them. The extremity of that portion of the artery connected with the sac, was much contracted, and completely filled by coagulum. The portion next to the heart was not in any degree contracted, and its cavity was open to the extremity, which was, in part only, filled by lymph. This lymph closed the orifice of the artery, except at one part, where there was an aperture that would receive the end of a probe between the lymph and the extremity of the vessel, and leading directly into the cavity of the latter. Five large branches, viz. the vertebral, internal mammary, cervicalis profunda, superior intercostal, and inferior thyroideal, arose from the artery between its origin and the ligature. These branches arose close together, and at a distance of only half an inch from the part where the artery had been tied. The right side of the heart was filled with blood; the lining of the thoracic aorta was partially thickened by a pulpy substance, deposited beneath it. Some small shreds of coagulable lymph were found on the pleura. The upper lobe of the left lung was observed to adhere to the pleura costalis, and when separated from it, I discovered that the posterior part of the tumour had penetrated the chest, so that the sac was here in contact with the pleura. The sac contained about a tea-cup full of recent coagulum, and some large masses of lamelated fibrine. The sternal extremities of the three first ribs were in a great part absorbed; the brachial artery was not in any degree contracted, and was quite pervious to its termination in the sac. Immediately below the sac, a large branch arose from the artery, almost equal in size to the trunk, and immediately divided into the infra-scapular and two circumflex arteries." 23.

The condition of parts, as ascertained by dissection, was unfavourable to an operation, and would probably have rendered it, even if successful at first, abortive in the end. We cannot but think that the success of an operation of this kind on a large artery mainly depends on the non-separation, to any extent, of the vessel from its connexions. If it were possible to pass a needle and thread round an artery without any separation at all, we think the success would be far greater than it now is. But as this cannot be done, we imagine the surgeon should endeavour to come as near to it as he can. We consider the above operation as very creditable to Mr. Mayo. The paper is written with modesty and perspicuity.

(To be continued in our next.)

